# Parkinson’s Disease Protects Against Smoking?

**DOI:** 10.1155/2004/516302

**Published:** 2005-01-27

**Authors:** Mohamed Farouk Allam, Michael J. Campbell, Amparo Serrano Del Castillo, Rafael Fernández-Crehuet Navajas

**Affiliations:** ^1^Department of Preventive Medicine and Public HealthFaculty of MedicineUniversity of CordobaCordobaSpain; ^2^Institute of General Practice & Primary CareUniversity of SheffieldSheffield S5 7AUUK

**Keywords:** Parkinson’s disease, smoking, systematic review, meta-analysis

## Abstract

Our aim was to estimate the pooled risk of current and former smoking for Parkinson’s disease (PD).We have reviewed all observational studies that evaluated the association between PD risk and smoking habit. Twenty six studies were identified: 21 case-control, 4 cohort and 1 cross-sectional. The cross-sectional study did not compare former with never smokers. These studies were carried out between 1968 and 2000.

There was an obvious protective effect of current smoking in the pooled estimate [risk estimate 0.37 (95% confidence interval 0.33 to 0.41)]. Former versus never smokers had pooled risk estimate of 0.84 (95% confidence interval 0.76 to 0.92). Current and former smoking do not, therefore, exert the same protective effect against PD so that it is unnecessary to postulate a biological mechanism through which smoking protects against PD. The results show that the reverse direction of causation is a more probable explanation, i.e. movement disorders of PD protect against smoking. Another explanation is that failure to develop strong smoking habits in early adult life might be a prodromal symptom of the disease and could perhaps be its first clinical manifestation.

## 1. Introduction

Numerous studies over the past several decades have demonstrated an inverse association between cigarette smoking and Parkinson's disease (PD), although clear explanations are lacking. The intriguing association was generally supported by many case-control and prospective cohort studies. However, recent case-control studies did not identify a clear protective effect and were in favor of the association reported in the other studies possibly being artefactual [8,11,32]. We also noted when reviewing studies supporting the inverse association that they showed quite a wide range of risk estimates.

Hypotheses concerning the relationship of smoking to lowered risk of PD, apart from those proposing real biological mechanisms to explain how smoking protects against PD, are various and independent. Previously advanced hypotheses have included: 1) selective mortality among smokers at early ages because of smoking's side effects and/or the selective genetic predisposition of smokers, which tends to lead to their early death [11,32]; 2) confounding, common life styles associated with smoking, such as coffee drinking, which was recently suggested to be associated with lower incidence of PD [8,32]; 3) reverse causation, according to which PD is associated with incomplete motor control that makes it less likely that PD patients will smoke or continue to smoke because of the motoric challenge posed when compared to people with normal levels of motor control [8,11,32]; 4) PD-associated personality differences, according to which persons predestined to get PD tend to be more passive, more introspective, more self-controlled, less likely to take risks and so tend to choose not to smoke [11].

The first two hypotheses could be excluded based on the results of cohort studies that adjusted their results

ISSN 0953-4180/04/$17.00 © 2004 – IOS Press and the authors. All rights reserved for age and possible confounding factors like coffee intake. Meanwhile, the latter two possibilities were not investigated sufficiently till now.

Comparing the risk of PD in current and former versus never smokers provides a fuller examination of the real association [21]. If current and former smoking exert the same protective effect, then it is acceptable to argue that smoking truly provides protection against developing PD. If, however, current smoking demonstrates a greater protective effect than former smoking, then a cause-and-effect bias is the explanation.

The aim of the present study was to estimate the pooled risk of current and former smoking for PD.

## 2. Materials and methods

### *2.1. Literature review*

Published observational studies on PD and cigarette smoking were identified through a comprehensive MEDLINE search (from 1966 to January 2001) and PsycLIT (from 1887 to January 2001) using a variety of Medical Subjects Headings and free text words: (SMOKING OR TOBACCO OR CIGARETTE) AND (PARKINSON OR PD) AND (CASE-CONTROL OR CASE-REFERENT OR RETROSPECTIVE OR COHORT OR FOLLOW-UP OR INCIDENT OR PROSPECTIVE OR EPIDEMIOLOGY). We conducted additional searches of Current Contents, Best Evidence, Nisc Mexico Biblioline, previous reviews, examination of cited reference sources and personal contact and discussion with several investigators expert in the field. Published case reports, studies evaluating other smoking habits, or smoking habits not stratified as current and former smokers were excluded. When two or more papers were based on an identical study, the paper that principally investigated the relationship between PD and smoking was used. We considered studies in all languages and no attempts were made to locate any unpublished study.

### *2.2. Data extraction*

We identified 26 published studies on the basis of our inclusion criteria [1–6,9,10,12,14,17–19,21,23,25,28–30,33,36,38,40,41,44,45]. A copy of each paper identified was obtained, and relevant data were abstracted by the first author (M.F.A.) for a quantitative overview. The type of risk estimate (i.e. relative risk, standardized mortality rate, odds ratio or prevalence odds ratio) and the country where the study was carried out were also ascertained. In case of discrepancies or when the information presented in a study was unclear, abstraction by a second reviewer (R.F.N.) was sought to resolve the discrepancy. All the included studies, except one, reported associations for current and former smokers. The remaining study reported only comparisons for current smokers [21].

### *2.3. Statistical methods for meta-analysis*

Data were abstracted from every study in the form of a risk estimate and its 95% confidence interval. When a risk estimate and its 95% confidence interval were not available from the article, we calculated unadjusted values from the published data of the article, using the Epi Info 6 computer program version 6.04d [13].

Risk estimate refers to relative risk or odds ratio. Relative risk compares the probability of an outcome (PD) among individuals who have been exposed to a given risk factor (smoking) to the probability of that outcome (control) among individuals who have not been exposed (never smoker). It means that the disease is relative risk times more likely to occur among those exposed to the risk factor than among those with no such exposure. Relative risk can be calculated only in a cohort or experimental study, meanwhile odds ratio can be calculated only in a case-control study [39].

Pooled risk estimate was obtained by weighing each study by the inverse variance of the effect measure on a logarithmic scale. This approach to pool the results assumes that the study populations being compared are similar and hence corresponds to a fixed effect analysis. The validity of pooling the risk estimates was tested (test of homogeneity) using a Chi square test [20].

A violation of this test implies that the studies being grouped differ from one another. In the presence of significant heterogeneity of the effect measure among studies being compared, we performed a random effect analysis that was based on the method described by DerSimonian and Laird (1986) [37]. The random effect analysis accounts for the interstudy variation. Because the test of homogeneity has low power, we reported the figures of the random effect analysis even with the absence of significant heterogeneity.

All statistical analysis for pooling the studies were performed on the STATA Statistical Software, release 7.0 (StataCorp. 2001).

Table 1
Studies evaluating the risk of PD in current versus never smokers

| Authors | Year of publication | Study type | Risk estimate | 95% confidence interval |
|---|---|---|---|---|
| Giroud-Benitez | 2000 | CS | 0.12 | 0.08–0.19 |
| Benedetti | 2000 | CC | 0.62 | 0.38–1.01 |
| Kuopio | 1999 | CC | 0.50 | 0.20–1.24 |
| Nelson | 1999 | CC | 0.22 | 0.12–0.40 |
| Fall | 1999 | CC | 0.17 | 0.06–0.43 |
| Chan | 1998 | CC | 0.52 | 0.26–1.01 |
| Hellenbrand | 1997 | CC | 0.26 | 0.18–0.39 |
| de Rijk | 1997 | C | 0.50 | 0.18–1.43 |
| Ben-Shlomo | 1996 | CC | 0.36 | 0.20–0.67 |
| Martyn | 1995 | CC | 0.49 | 0.26–0.91 |
| Doll | 1994 | C | 0.75 | 0.38–1.46 |
| Grandinetti | 1994 | C | 0.24 | 0.12–0.48 |
| Mayeux | 1994 | CC | 0.20 | 0.10–0.50 |
| Sasco | 1990 | CC | 0.51 | 0.23–1.13 |
| Zayed | 1990 | CC | 0.10 | 0.00–0.83 |
| Hofman | 1989 | CC | 0.70 | 0.40–1.40 |
| Rajput | 1987 | CC | 0.46 | 0.23–0.92 |
| Granerus | 1987 | CS | 0.77 | 0.33–1.81 |
| Cazzato | 1985 | CC | 0.12 | 0.05–0.31 |
| Ogawa | 1984 | CC | 0.58 | 0.38–0.87 |
| Godwin-Austen | 1982 | CC | 0.48 | 0.32–0.71 |
| Marttila | 1980 | CC | 0.38 | 0.23–0.62 |
| Rogot | 1980 | C | 0.32 | 0.24–0.43 |
| Kessler | 1972 | CC | 0.42 | 0.25–0.70 |
| Kessler | 1971 | CC | 0.46 | 0.32–0.66 |
| Nefzger | 1968 | CC | 0.31 | 0.18–0.55 |
| Pooled risk estimate | | | 0.37 | 0.33–0.41 |

CC: Case–control study.
CS: Cross-sectional study.
C: Cohort study.

## 3. Results

We localized 63 observational studies that evaluated the association between PD risk and cigarette smoking. Only 26 studies compared PD rates in current and never smokers, and of these studies four were prospective studies. The 26 studies included one cross-sectional study that did not compare former with never smokers. Most of the studies were published in English and only five studies were published in French, Spanish, Italian, Swedish and Japanese. The studies represented different populations of all continents except Africa and Australia. The results of these studies were published between 1968 and 2000.

### *3.1. The risk of PD in current versus never smokers*

The pooled risk estimate of current versus never smokers demonstrated a one-third risk of PD in current smokers [risk estimate 0.37 (95% confidence interval 0.33 to 0.41)]. The chi-square value of the homogeneity test was 72.6 with p value $<0.001$, denoting heterogeneity across the pooled twenty-six studies (Table 1). Pooled analysis applying the random effect model was 0.37 (95% confidence interval 0.31 to 0.45).

### *3.2. The risk of PD in former versus never smokers*

Former versus never smokers had pooled risk estimate of 0.84 with 95% confidence interval 0.76 to 0.92. The homogeneity test of the twenty-five studies had a p value of 0.24, indicating homogenous results of the pooled studies (Table 2). Pooled analysis applying the random effect model was 0.83 (95% confidence interval 0.75 to 0.92).

## 4. Discussion

Perhaps one of the most important questions posed by the neurobiology of aging concern the pathogenic mechanisms in PD. Although the aetiology of PD is still unknown, increasing evidence supports the hypothesis that environmental factors may contribute to its occurrence. Our meta-analysis was intended to check the role of an environmental factor, smoking habit, known

Table 2
Studies evaluating the risk of PD in former versus never smokers

| Authors | Year of publication | Study type | Risk estimate | 95% confidence interval |
|---|---|---|---|---|
| Benedetti | 2000 | CC | 1.14 | 0.41–3.15 |
| Kuopio | 1999 | CC | 1.08 | 0.66–1.76 |
| Nelson | 1999 | CC | 0.99 | 0.75–1.29 |
| Fall | 1999 | CC | 0.82 | 0.44–1.51 |
| Chan | 1998 | CC | 0.91 | 0.62–1.33 |
| Hellenbrand | 1997 | CC | 0.92 | 0.69–1.23 |
| de Rijk | 1997 | C | 0.26 | 0.09–0.78 |
| Ben-Shlomo | 1996 | CC | 0.73 | 0.47–1.14 |
| Martyn | 1995 | CC | 0.61 | 0.40–0.94 |
| Doll | 1994 | C | 1.10 | 0.60–2.02 |
| Grandinetti | 1994 | C | 0.50 | 0.27–0.93 |
| Mayeux | 1994 | CC | 0.90 | 0.50–1.60 |
| Sasco | 1990 | CC | 0.77 | 0.35–1.69 |
| Zayed | 1990 | CC | 1.75 | 0.74–4.18 |
| Hofman | 1989 | CC | 0.50 | 0.30–1.00 |
| Rajput | 1987 | CC | 1.10 | 0.60–2.03 |
| Granerus | 1987 | CS | 0.99 | 0.42–2.36 |
| Cazzato | 1985 | CC | 1.02 | 0.51–2.04 |
| Ogawa | 1984 | CC | 0.74 | 0.30–1.67 |
| Godwin-Austen | 1982 | CC | 0.80 | 0.56–1.15 |
| Marttila | 1980 | CC | 1.04 | 0.73–1.49 |
| Rogot | 1980 | C | 0.70 | 0.52–0.94 |
| Kessler | 1972 | CC | 0.54 | 0.33–0.88 |
| Kessler | 1971 | CC | 0.92 | 0.67–1.26 |
| Nefzger | 1968 | CC | 0.68 | 0.37–1.21 |
| Pooled risk estimate | | | 0.84 | 0.76–0.92 |

CC: Case–control study.
CS: Cross-sectional study.
C: Cohort study.

for its harmful effects generally, but offering a possible protection against PD, mediated by biological mechanism.

Before reaching conclusions based on the present results, it is necessary to consider a number of potential objections to our procedures. Methodological concerns include limitations in the quality of the primary data, as the usefulness of meta-analysis is largely dependent on the quality of the studies used. Combining randomized controlled trials provides more evidence, but is clearly impossible in studying the aetiology of a chronic disease as in our case [43].

Reviewing the literature, there have been four systematic and narrative reviews that investigated the smoking habit/PD association, but our comprehensive search has identified more studies than previous reviews [11,15,24,32]. These reviews missed last follow-up or even complete studies not cited by MEDLINE. The results of Rogot and Murray (1980) of 16 years follow-up on US Male Veterans have never been included before [12]. The Rotterdam, a population-based cohort study, was not identified by other reviewers. This study reported results of incident idiopathic PD cases after well-established criteria for case identification [28]. Recently, two review studies [24,32], one of them a quantitative review [32], reported results of 20 years' observations on Male British Doctors, although 40 years' observations were published in 1994 [38].

Also, and of note, quantitative reviews are usually restricted to studies published in English. This raises concern regarding the external validity of their results [26].

Our meta-analysis has included studies published in six languages. The study of Zayed et al. (1990), published in French, has never been included before in other quantitative review [23].

The pooled studies in our meta-analysis were well designed and carefully executed. However, they varied with regard to study design, size, quality of diagnosis and measure of exposure. We think that pooling of this heterogeneous set of studies adds useful information to that provided by individual studies of the highest quality, such as the Male British Doctors or the Honolulu Heart Study.

Our meta-analysis showed that there was an obvious protective effect of current smoking in the pooled estimate [risk estimate 0.37 (95% confidence interval 0.33 to 0.41)]. This means that current smoking is inversely associated with the risk of PD, in concordance with the results of several previous studies [11,32]. However, the homogeneity test was very highly significant, which proves the heterogeneity of the results of pooled studies. The heterogeneous and contradictory results could not describe a real biological protective mechanism and calls for a consideration of possible biases.

Pooled risk estimate between former smoking and PD was of 0.84 (95% confidence interval 0.76 to 0.92). The homogeneity test had a non-significant p value, denoting homogeneity of the pooled results. These consistent results should be considered more conclusive and precise.

It is of note that the four cohort studies included have demonstrated inconsistent results in the subgroup analysis. Meanwhile, US Veterans Study, Male British Doctors Study and Honolulu Heart study results showed that former smokers are at intermediate risk between current and never smokers [1,12,38]. The Rotterdam Study showed a higher risk of idiopathic PD among current smokers [28]. This contradictory result was based on only 34 incident cases during a three-year follow-up. The Rotterdam study is ongoing since 1990 and future analysis with new incident cases should help elucidate this contradictory result.

Also of note, the retrospective studies that evaluated the risk of PD in current versus never smokers have not demonstrated a clear protective effect with pooled odds ratio of 0.87 (95% CI 0.79 to 0.96). In addition, many retrospective studies have shown a clear inverse association in comparisons of current versus never smokers. The same studies in comparisons of former with never smokers reported odds ratios with 95% confidence intervals that overlapped unity, and so were inconsistent with the protective effect hypothesis [2,14,17,18,23,25, 30,33,36,40,41,44,45].

PD is an acquired disease, associated with toxic-environmental exposure 20 to 40 years before the appearance of the first symptoms [16,22]. Evaluation of the relation between tobacco smoking and PD risk, comparing former smokers with non-smokers, should be more appropriate than any other comparison. The low frequency of current smokers among PD patients, in turn, cannot only be attributed to the possible protective effect of tobacco smoking. Systematic biases, like reverse causation and PD-associated personality difference, could be the explanation [11,28].

Our meta-analysis shows that the reverse causation bias is a possible explanation since the pooled analysis of former smokers had a relative risk much closer to unity, and it is more probable that movement disorders of PD protect against smoking.

It is logical that patients with neurological disease associated with movement disorders give up smoking. Patients with PD may stop smoking after the onset of the disease because of physiological reasons or psychological dislike, and this affects the validity of the results of the case-control studies [11,15]. By extension, the first symptoms of PD appear many years before its diagnosis, with loss of fine movements, and these persons might choose not to smoke.

Another explanation is that failure to develop strong smoking habits in early adult life might be a prodromal symptom of the disease; it could be the first clinical manifestation [11]. The lower incidence of alcoholism in PD, noted since 1966, suggests an early behavioral expression of the disease [31]. If so, there could be something different about PD patients many years before the onset of extrapyramidal symptoms, probably as early as late adolescence or early adult years, when smoking and drinking habits tend to be acquired [11, 16,22].

It has been suggested that subjects showing novelty-seeking behavior and addiction have higher dopamine levels [27,31]. Recent results from the Rotterdam study show that cigarette smoking, alcohol drinking and coffee consumption have a reduced risk of PD, and the trend towards lower risks is associated with higher exposures. Since three different addictive agents show this same trend, the authors explained their findings by the higher endogenous dopamine levels in subjects showing novelty-seeking behavior and addiction [35].

In concordance with our hypothesis, controlled clinical trials have failed to demonstrate a beneficial effect of nicotine consumption in PD. Nicotine chewing gum and transdermal nicotine patches have been used as an add-on treatment for the symptoms of PD. However, both forms of nicotine administration had no significant effects [7,34].

In conclusion, stratification of the smoking habit, current and former smoking, is a promising explanation for the controversial association between PD and tobacco smoking, and calls for further studies.
